# Effect of the Layer Sequence on the Ballistic Performance and Failure Mechanism of Ti6Al4V/CP-Ti Laminated Composite Armor

**DOI:** 10.3390/ma13173886

**Published:** 2020-09-02

**Authors:** Hong Yu, Qunbo Fan, Xinjie Zhu

**Affiliations:** 1 School of Materials Science and Engineering, Beijing Institute of Technology, Beijing 100081, China; yuhong202106@163.com (H.Y.); zhuxinjielyw@163.com (X.Z.); 2 National Key Laboratory of Science and Technology on Materials under Shock and Impact, Beijing 100081, China; 3 Beijing Institute of Technology Chongqing Innovation Center, Chongqing 401147, China

**Keywords:** laminated composite armor, energy absorption, ballistic performance, numerical simulation

## Abstract

The effect of the layer sequence on the ballistic performance of Ti6Al4V (35 mm)/CP-Ti (5 mm) laminated composite armor, against a 12.7 mm armor piercing projectile, was systematically investigated, both experimentally and computationally. By introducing the Johnson–Cook constitutive model and fracture criterion, the penetrating process of the composite plate was well-simulated. Furthermore, the influence of the layer sequence on the ballistic performance and failure mechanism of the composite plate was evaluated from the perspective of energy absorption and the stress distribution. Numerical simulation results of the macro morphology and penetration depth agreed well with the experimental results. The results showed that the energy absorption histories of each layer and stress distribution of the composite plate were found to be significantly affected by the arrangement sequence. The ballistic performance of Ti6Al4V/CP-Ti was far superior to that of CP-Ti/Ti6Al4V because more energy was absorbed in the early stage of the penetration process, thereby reducing the damage to the rear face. Further studies showed that the first principal stress in both structures was radially distributed in space, but was mainly concentrated at the rear face when the CP-Ti was placed at the front. Therefore, this stress induced cracking and failure in that region and, consequently, lowered the overall ballistic performance.

## 1. Introduction

With the ongoing threat of warheads, the ballistic resistance of monolithic plates has become increasingly unable to resist these threats [1]. Therefore, laminated composite armor consisting of the same or different materials has aroused the interest of researchers, due to its excellent overall performance. These armors combine the mechanical properties of each layer and structural effects, thereby delivering optimal ballistic performances and representing a suitable alternative to monolithic metallic plates.

The effects of structural parameters (e.g., the number of layers [2,3,4,5,6,7,8,9] and layer spacing [5,10,11,12,13]) on the ballistic performance of laminated composite armor have been extensively investigated. Additionally, laminated metal armor plates consisting of several kinds of metal layers have been further studied. Wang [14] and Zhou [1] investigated laminate plates consisting of steel and aluminum by a ballistic experiment and simulation. They found that the ballistic performance is closely related to the thickness ratio of steel and aluminum. Li et al. [15] conducted several ballistic impact tests on a 7A52/7B52 aluminum laminate plate, and found that the failure modes of the layers were significantly different under the same impact condition. Further studies have showed that the ballistic performance and failure mechanism of composite armors vary with the sequence of layers. Teng et al. [16] investigated the ballistic resistance of a double-layer composite plate via a numerical simulation. They found that the failure mode of the composite target plate changes from shear plugging to tensile fracture when the ductile material is placed at the front. This resulted in increased energy absorption of plastic deformation and an improved ballistic performance. Flores-Johnson et al. [3] investigated a double-layered metallic plate consisting of either steel or aluminum or a combination of these materials. The results revealed that double-layered plates with a thin aluminum front plate and a thick steel back plate exhibit a greater resistance than multi-layered steel plates with a similar areal density. Nevertheless, as reported by Awerbuch et al. [17], a shield with a steel frontal layer and an aluminum back layer was more effective than a shield with the reverse ordering of layers. Similarly, Buchar et al. [18]. found that dual hardness laminated armor, where the hardness of the front layer is higher than that of the back layer, exhibited an excellent resistance against the impact of an armor piercing (AP) projectile. In addition, the ballistic performance improved when increasing the thickness of the high hardness steel.

Previous studies have mainly focused on laminated composite armor composed of steel and aluminum. However, owing to its relatively high density, steel offers no advantage for weight reduction and, despite its low density, aluminum has poor mechanical properties. In recent years, titanium alloys have been widely investigated for armor applications due to attributes such as their high specific strength, excellent corrosion resistance, and non-magnetic properties [19,20,21,22]. Monolithic titanium plates are prone to shear plugging [23,24] and back spalling [19] during impact, and the use of titanium alloys to produce composite plates has attracted increasing attention. McLaurin [25] systematically studied a titanium laminated plate, and the results showed that targets with multiple layers (laminate) performed better than monolithic targets of the same thickness. Perkins et al. [26] successfully produced and tested titanium/titanium dual hardness composite armor. The results revealed that the front layer of high hardness titanium could shatter both 7.62 and 12.7 mm AP projectiles, and cracking or delamination can be avoided via using appropriate producing processes. Fras et al. [27] prepared titanium/aluminum laminated composite armor consisting of a Ti6Al4V frontal plate, an AA2519 rear plate, and an AA1050 interlayer. In terms of the area density, this armor was far superior to the steel armor plate. Bruchey [28] produced a Ti6Al4V/CP-Ti laminated composite armor plate with a ballistic performance that was 5–10% better than that of a single Ti6Al4V weight equivalent plate.

Researchers have discussed the influence of the layer sequence on the ballistic performance of laminated composite armor, but a general consensus regarding this influence is lacking [16,17]. Although some studies have explained the deformation and failure modes of the laminated composite armor, few studies have focused on and quantitatively analyzed the energy absorption process and stress distribution. In addition, studies on the ballistic resistance of titanium have received increasing attention, whereas studies on laminated composite armor composed of titanium are relatively rare. In this study, we fabricated a Ti6Al4V/CP-Ti laminated composite armor plate by means of explosive welding, and the composite plate, with two different arrangement sequences, was penetrated by a 12.7 mm armor piercing incendiary (API) projectile. The Johnson–Cook constitutive model and fracture criterion were adopted to model the laminated composite plate. The penetration process was simulated using the non-linear finite element program ANSYS/LS-DYNA (V4.2, LSTC). The simulated macro morphology and damage behavior along the penetration channel were assessed and compared with the experimental results. Moreover, the effect of the layer sequence on the ballistic performance of the laminated composite plate was analyzed in detail from the perspective of energy absorption. Meanwhile, the stress distribution of the target plate in different layer sequences was deeply studied by a numerical simulation, in order to reveal the damage mechanism of the composite plate.

## 2. Experimental Procedure

**Figure 1:**
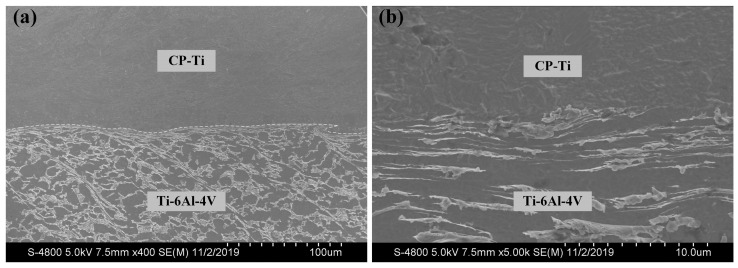
Microstructure of the Ti6Al4V/CP-Ti interface. (**a**) Scanning electron microscope image and (**b**) high magnification image.

In our previous experiments, we tested Ti6Al4V/CP-Ti laminated plates which were boned by polyurethane adhesive, and the results showed that the laminated plate consisting of 35 mm Ti6Al4V and 5 mm CP-Ti had the best ballistic performance and a lower weight compared to other titanium laminated structures against a 12.7 mm API projectile. Therefore, explosive welding was adopted to bond the Ti6Al4V (35 mm)/CP-Ti (5 mm) plates to improve the bonding strength. Explosive welding enables metallurgical bonding on the entire junction surface without interface oxidation and is therefore considered a promising solid-state welding method [29]. The dynamite was prepared with an ammonium nitrate fuel oil (ANFO) mixture (detonation velocity: 2300 m/s). The commercial Ti6Al4V and CP-Ti were used as the parent plate and flyer plate, respectively (the corresponding mechanical properties are shown in Table 1). Subsequently, the Ti6Al4V (35 mm)/CP-Ti (5 mm) laminated composite plate was obtained by means of parallel explosive welding and then planished using a hydraulic press. The cross-sectional microstructure near the Ti6Al4V/CP-Ti interface (see Figure 1) revealed that Ti6Al4V and CP-Ti were well-bonded and the interface was free of inclusions and cavities.

**Figure 2:**
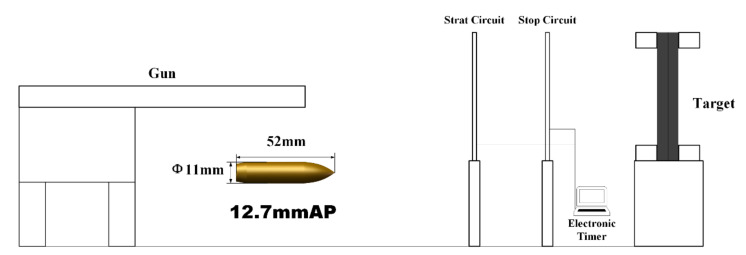
Schematic of the ballistic experiment.

A Ti6Al4V/CP-Ti laminated target with dimensions of 300 × 300 mm^2^ was fixed on a platform 50 m away from the ballistic gun. The target board was vertically placed and fixed around this target with a bracket to ensure that the back of the board was free and unsupported. The projectile used in the experiment was a 12.7 mm API fired from a standard ballistic gun (see Figure 2 for a schematic of the experimental setup). The impact velocities of the projectile were measured by testing the time interval between the projectile passing through two special aluminum foils (the Start circuit and Stop circuit in Figure 2) placed 1 m apart. The target plates were impacted vertically (projectile impact speed: 800 m/s). Two schemes were considered in the ballistic tests: (1) The Ti6Al4V was placed at the front (hereafter, this configuration is referred to as Ti64/CPTi), and (2) the CP-Ti was placed at the front (hereafter, this configuration is referred to as CPTi/Ti64). After the ballistic test, the target plates were recycled and machined into halves through the mid-section of the crater for further analysis. 

## 3. Numerical Simulation

### 3.1. Finite Element Model

**Figure 3:**
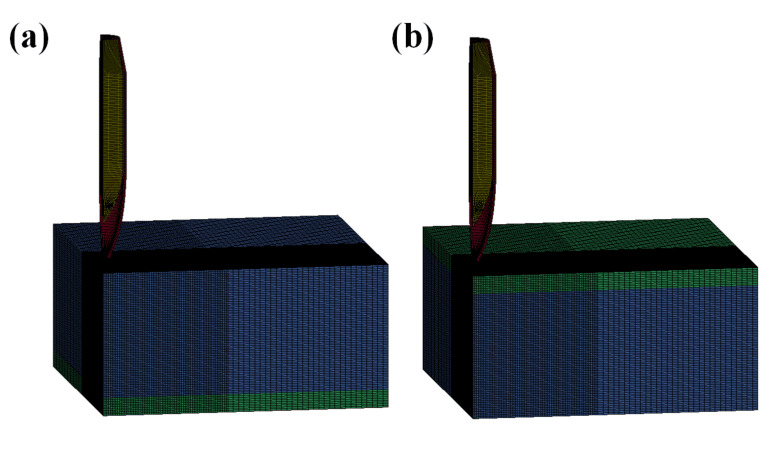
FEM model of the calculation: (**a**) Ti64/CPTi and (**b**) CPTi/Ti64.

**Figure 4:**
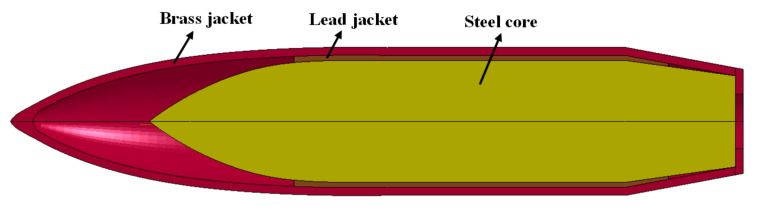
Structural details of the projectile.

The Lagrange method of ANSYS/LS-DYNA was used for the numerical simulation. The problem was considered symmetric and, hence, a quarter of the finite element model (see Figure 3, the FEM model’s perspective was rotated in order to show the structural components clearly) was established, in order to reduce the computational costs. Symmetric constraints were applied to the symmetry plane and the boundaries of the target plate were fully constrained. The structural details of the 12.7 mm API projectile with an ogival nose are shown in Figure 4. It consisted of a steel core, lead jacket, and brass jacket, with a total weight of 40.0 g. The projectile was given an initial velocity of 800 m/s, which was the same in the corresponding experiments. SOLID164 elements with eight nodes were used for meshing. A mesh sensitivity study was performed and four different element sizes of 1, 0.75, 0.5, and 0.25 mm were used for numerical simulation. The results indicated that the model converged toward a limit solution when the element size was less than 0.75 mm. In order to reduce the computational time and ensure the calculation accuracy, the element size in the impact region was selected to be 0.5 mm. Correspondingly, 33,044 and 800,000 elements (in total) were employed for the projectile and composite plate, respectively.

### 3.2. Material Models

In this study, the three components of the projectile (steel core, lead jacket, and brass jacket) were modeled using the Cowper–Symonds model [30]. The constitutive equation of the model is given as follows:

(1) $$\sigma_{y}=\sigma_{0}\left[1+{\left(\dot{\varepsilon }/C\right)}^{1/P}\right],$$

where $\sigma_{y}$ is the dynamic flow stress, $\sigma_{0}$ is the quasi-static yield strength, $\dot{\varepsilon }$ is the uniaxial strain rate, and C and P are the strain rate parameters. Table 2 shows the material properties of the projectile components [9]. E, ρ, υ, and G are the Young’s modulus, density, Poisson’s ratio, and tangent modulus, respectively.

The Johnson–Cook material constitution [31] was employed for modeling the Ti6Al4V and CP-Ti. The constitutive relations were implemented in LS-DYNA as *MAT_JOHNSON_COOK [32], where the governing equation involving the strain, strain rate, and temperature, is given as follows:

(2) $$\sigma_{y}=\left[A+B\varepsilon_{p}^{n}\right]\left[1+Cln\dot{\varepsilon_{p}^{*}}\right]\left[1-T_{H}^{m}\right],$$

where $\sigma_{y}$ is the equivalent stress, $\varepsilon_{p}$ is the effective plastic strain,$\;\dot{\varepsilon_{p}^{*}}$ is the normalized effective plastic strain rate, and $T_{H}$ is the homologous temperature. A, B, C, n, and m represent the static yield stress, hardening parameter, strain rate parameter, hardening index, and temperature index, respectively. The Johnson–Cook material constants of Ti6Al4V [33] and CP-Ti [34] used for the simulation are shown in Table 3. 

The Johnson–Cook damage model was adopted to model the Ti6Al4V and CP-Ti. The damage model can be expressed as

(3) $$\varepsilon_{f}=\left[D_{1}+D_{2}\exp\left(D_{3}\sigma^{*}\right)\right]\left[1+D_{4}ln\dot{\varepsilon_{p}^{*}}\right]\left[1+D_{5}T_{H}\right],$$

where $\varepsilon_{f}$ is the equivalent fracture strain; $\sigma^{*}$ is the stress triaxiality; and D_1_, D_2_, D_3_, D_4_, and D_5_ are material constants. The damage parameter $D=\sum \left(\Delta \varepsilon /\varepsilon_{f}\right)$ takes values between 0 and 1, and failure of the elements is assumed to occur when D = 1. The parameters used in the Johnson–Cook damage model are listed in Table 3.

The interaction between the projectile and the target plate was described using the *ERODING_SINGLE_SURFACE [32] contact algorithm. Moreover, the *AUTOMATIC_SURFACE_TO_ SURFACE _TIEBREAK [32] contact algorithm was used to model the combination of the Ti6Al4V and CP-Ti layers.

## 4. Results and Discussion

### 4.1. Penetration Results

**Figure 5:**
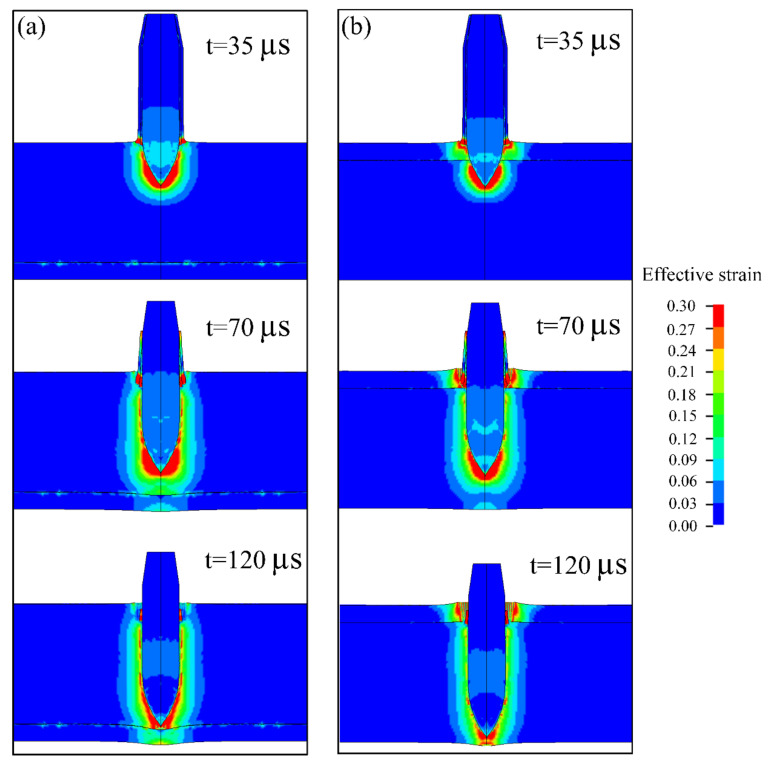
The strain distribution of the projectile and plate during the penetration process: (**a**) Ti64/CPTi and (**b**) CPTi/Ti64.

Figure 5 shows the strain distribution of the projectile and plate during the penetration process. At t = 35 µs, the steel core penetrated the target plate, and the brass jacket and lead jacket developed serious plastic deformation and failed quickly when interacting with the target plate. Severe plastic deformation with the effective strain of 0.3 occurred in the titanium alloy near the tip of the steel core in both schemes. For CPTi/Ti64, the plastic deformation of the CP-Ti layer was also considerable. At t = 70 µs, the brass jacket and lead jacket penetrated the target plate, thus enlarging the crater on the front of the target plate of both Ti64/CPTi and CPTi/Ti64, but almost half of the brass and lead had failed due to severe deformation. At t = 120 µs, the projectile had been stopped. The brass jacket and lead jacket were totally ripped off and most of them failed, causing a larger deformation on the target plate. However, the tip of the steel core experienced small plastic deformations during penetration. Although Ti6Al4V produces large plastic deformation in both schemes, the plastic deformation of CP-Ti in Ti64/CPTi is relatively small compared with CPTi/Ti64.

**Figure 6:**
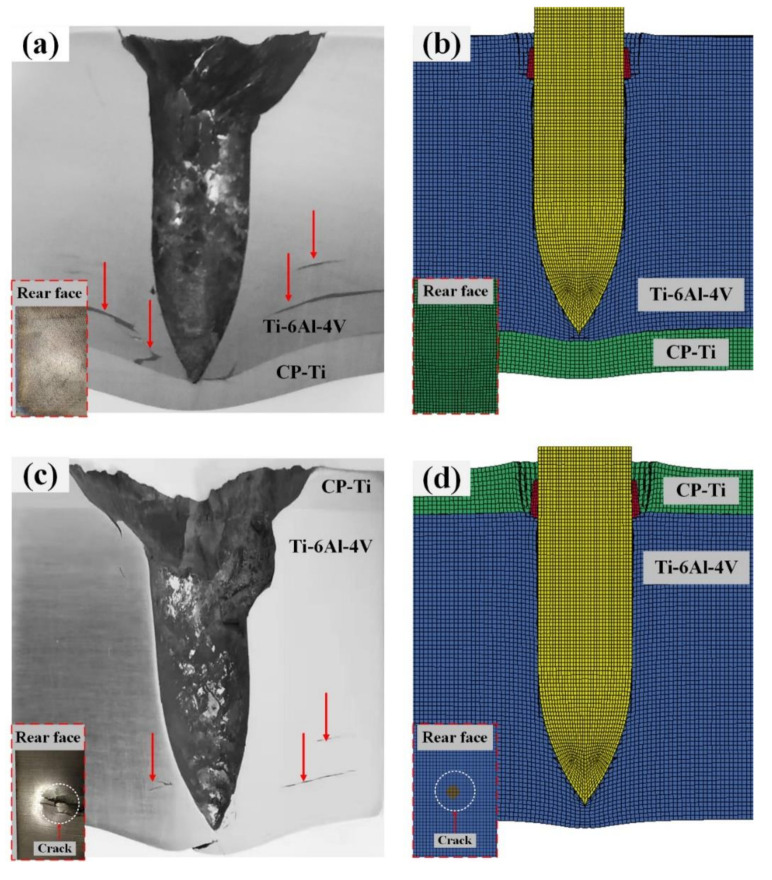
Macro morphologies of the cross-section along the penetration channel of the Ti6Al4V/CP-Ti laminated composite armor: (**a**,**c**) Experimental results and (**b**,**d**) simulation results of the Ti64/CPTi plate and CPTi/Ti64 plate, respectively.

Figure 6 shows the experimental and simulated macro morphologies of the cross-section along the penetration channel of the Ti6Al4V/CP-Ti laminated plates. The damage to the rear face of the target plate is shown in the lower left inset. As is shown in Figure 6a, lamellar tearing occurred in the Ti6Al4V that was indirectly connected to the penetration channel, and the rear face of the Ti64/CPTi plate underwent bulging without fracturing. Similarly, the numerical simulation results of the Ti64/CPTi plate (Figure 6b) revealed that bulging of the CP-Ti layer occurred without the deletion of elements. The lamellar tearing generated in the CPTi/Ti64 plate was less extensive and narrower than that observed for the Ti64/CPTi plate (see Figure 6c). However, severe cracks were generated on the back of the target plate. As revealed by the numerical simulation results of the CPTi/Ti64 plate (Figure 6d), element deletion occurred on the rear face of the target plate, that is, cracks were generated. Therefore, the macro morphology shown by simulations closely corresponded to the experimental results. A close correspondence (relative error: 5.3%) was also obtained for the experimentally determined penetration depths in the target plate and the numerically simulated depths, as shown in Table 4. In general, the Ti64/CPTi plate exhibited a better ballistic resistance (than the CPTi/Ti64 plate) because the damage was concentrated in the interior of the composite plate. This prevented cracking in the rear face, thereby ensuring overall structural integrity. Figure 5 shows the strain distribution of the projectile and plate during the penetration process. At t = 35 µs, the steel core penetrated the target plate, and the brass jacket and lead jacket developed serious plastic deformation and failed quickly when interacting with the target plate. Severe plastic deformation with the effective strain of 0.3 occurred in the titanium alloy near the tip of the steel core in both schemes. For CPTi/Ti64, the plastic deformation of the CP-Ti layer was also considerable. At t = 70 µs, the brass jacket and lead jacket penetrated the target plate, thus enlarging the crater on the front of the target plate of both Ti64/CPTi and CPTi/Ti64, but almost half of the brass and lead had failed due to severe deformation. At t = 120 µs, the projectile had been stopped. The brass jacket and lead jacket were totally ripped off and most of them failed, causing a larger deformation on the target plate. However, the tip of the steel core experienced small plastic deformations during penetration. Although Ti6Al4V produces large plastic deformation in both schemes, the plastic deformation of CP-Ti in Ti64/CPTi is relatively small compared with CPTi/Ti64.

### 4.2. Energy Analysis of the Penetration Process

During the penetration process, the target plate absorbs the kinetic energy of the projectile. The projectile was effectively stopped in both schemes. Therefore, assuming that other factors (such as the transformation of the kinetic energy into thermal energy) can be ignored, most of the initial projectile kinetic energy was absorbed by the composite target plate. In this work, the energy absorption of the target plate was quantitatively evaluated via numerical simulations. The elements can be classified as eroded elements and uneroded elements, and the total energy *E_total_* is given as follows:

(4) $$E_{total}=E_{eroded}+E_{uneroded},$$

(5) $$E_{eroded}=E_{k\_eroded}+E_{i\_eroded},$$

(6) $$E_{uneroded}=E_{k\_uneroded}+E_{i\_uneroded},$$

where *E_k_eroded_* and *E_k_uneroded_* are the kinetic energy values of eroded elements and uneroded elements, respectively. *E_i_eroded_* and *E_i_uneroded_* are the internal energy values of eroded elements and uneroded elements, respectively.

**Figure 7:**
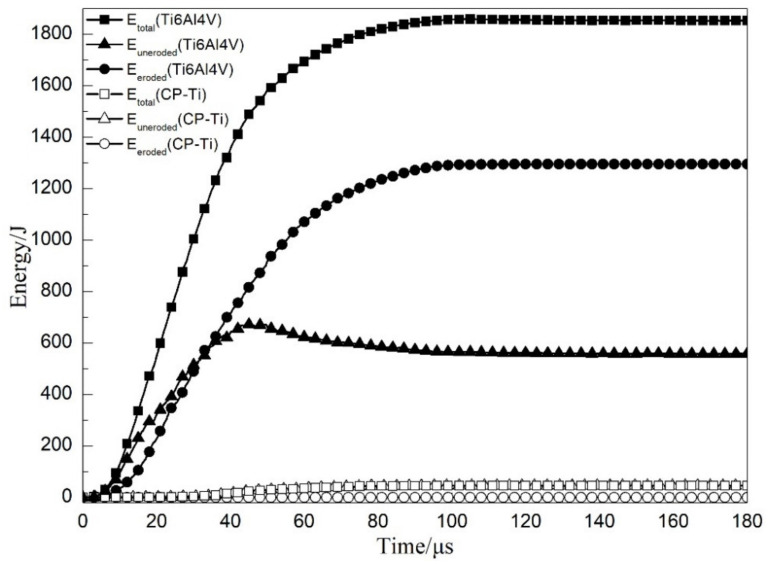
Energy absorption history curve of Ti6Al4V and CP-Ti subjected to the penetration process of Ti64/CPTi.

Figure 7 shows the energy absorption history curve obtained for layers of the Ti64/CPTi laminated composite plate subjected to the penetration process (0–180 μs). The total energy absorbed by the Ti6Al4V layer *E_total_* (Ti6Al4V) increased linearly in the early stage, and flattening of the curve began after 41 μs. The energy absorbed by the eroded elements of the Ti6Al4V layer *E_eroded_* (Ti6Al4V) was compared with the energy absorbed by the un-eroded elements of the layer *E_uneroded_* (Ti6Al4V). This comparison revealed that, compared with the eroded elements, the un-eroded elements absorbed more energy in less than 33 μs. However, with a continuous increase in the number of the eroded elements, *E_eroded_* (Ti6Al4V) dominated after 33 μs. This indicated that the energy absorbed by the target plate mainly depended on the plastic deformation of the Ti6Al4V in the early stage of the penetration process. Similarly, most of the energy absorption due to the failure of the Ti6Al4V occurred after 33 μs. Values of 1295 and 560 J were obtained for the energy absorption of the Ti6Al4V eroded elements and the Ti6Al4V un-eroded elements, respectively. For the 0–33 μs stage, there was no transfer of Ti6Al4V deformation to the CP-Ti layer and, hence, this layer absorbed almost no energy in this period. After 33 μs, the Ti6Al4V layer started to interact with the CP-Ti layer, thereby promoting the deformation and energy absorption of CP-Ti. CP-Ti remained almost damage-free during the entire penetration process of Ti64/CPTi (as shown in Figure 5a,b) and, hence, the eroded element energy absorption of the CP-Ti layer *E_eroded_* (CP-Ti) was 0 J. Therefore, the curve corresponding to the CP-Ti layer total energy absorption *E_total_* (CP-Ti) overlapped with the curve corresponding to the uneroded element energy absorption *E_uneroded_* (CP-Ti). This result revealed that the CP-Ti layer only absorbs energy (energy absorption: 45 J) by means of plastic deformation.

**Figure 8:**
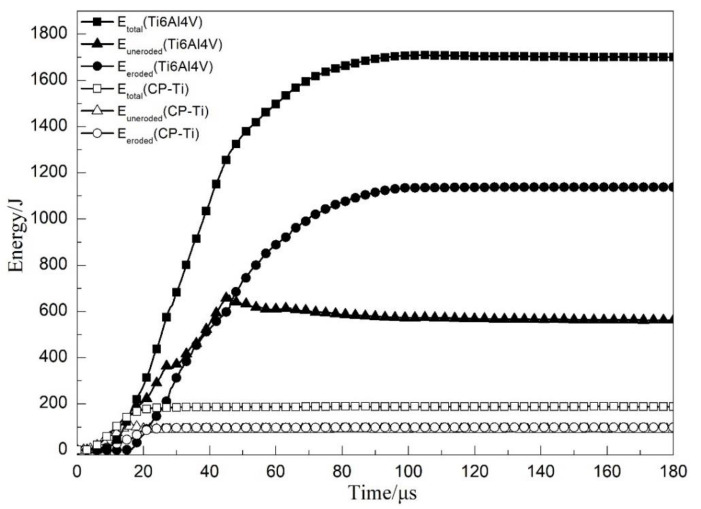
Energy absorption history curve of Ti6Al4V and CP-Ti during the penetration process of CPTi/Ti64.

The energy absorption history curve of each layer involved in the penetration process of the CPTi/Ti64 laminated composite armor plate is shown in Figure 8. From 0 to 23 μs, the projectile nose maintained contact with the CP-Ti layer and the entire projectile head then penetrated the layer, leading to continuous deformation and the failure of CP-Ti. Energy absorption values of 101 and 98 J were obtained for the eroded elements of CP-Ti *E_eroded_* (CP-Ti) and un-eroded elements of CP-Ti *E_uneroded_* (CP-Ti), respectively. After 23 μs, the projectile head perforated the CP-Ti layer completely, and the CP-Ti total absorbed energy *E_total_* (CP-Ti) became constant because this layer underwent no further deformation or failure. Subsequently, the projectile nose reached the Ti6Al4V layer (at 11 μs), and *E_total_* (Ti6Al4V) increased sharply due to the large deformation and failure of the Ti6Al4V. Energy absorption values of 1135 and 566 J were then obtained for the Ti6Al4V eroded elements *E_eroded_* (Ti6Al4V) and the un-eroded elements *E_uneroded_* (Ti6Al4V), respectively.

**Figure 9:**
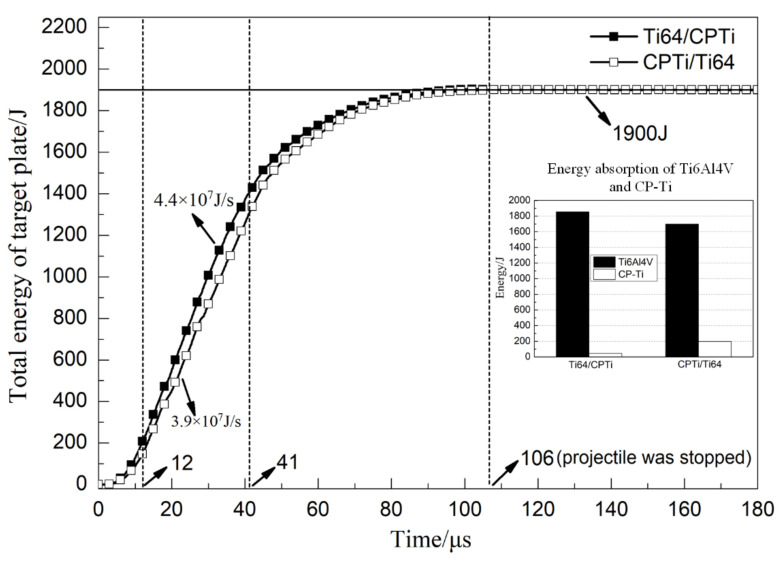
Total energy absorption curve of Ti64/CPTi and CPTi/Ti64 laminated composite armor plates.

Figure 9 shows the total energy absorption history curve of Ti64/CPTi and CPTi/Ti64. As shown in the figure, the curves in the two schemes exhibited the same trend: A rapid increasing stage (0–12 μs); a linear increasing stage (12–41 μs); and a slow increasing stage (41–106 μs). The energy absorption rate in both schemes was calculated from the slope of the linear stage. The results revealed that the energy absorption rate of Ti64/CPTi (4.4 × 10^7^ J/s) was higher than that of CPTi/Ti64 (3.9 × 10^7^ J/s). This indicated that more energy was absorbed in the early stage (than in the late stage) of the penetration process when the Ti6Al4V was placed on the striking face. Therefore, the fracture induced on the rear face of the laminated armor plate was alleviated. A total energy of 1900 J was absorbed by the target plate in each scheme. However, the differences between the energy absorption rates of Ti64/CPTi and CPTi/Ti64 in the early stage led to significant differences between the energy absorption distributions. As shown in the inset of Figure 9, the Ti6Al4V total energy absorption values of Ti64/CPTi and CPTi/Ti64 (1855 and 1701 J) accounted for 97.6% and 89.5%, respectively, of the total absorbed energy. The Ti6Al4V layer absorbed more energy during Ti64/CPTi penetration (than during CPTi/Ti64 penetration), so more lamellar tearing occurred in Ti6Al4V (as shown in Figure 6a,c). Therefore, the higher strength Ti6Al4V layer absorbed more kinetic energy as the striking face, while the CP-Ti layer maintained the structural integrity as the higher ductility back layer, thereby inhibiting crack propagation on the rear face.

### 4.3. Stress Analysis of the Target Plate

**Figure 10:**
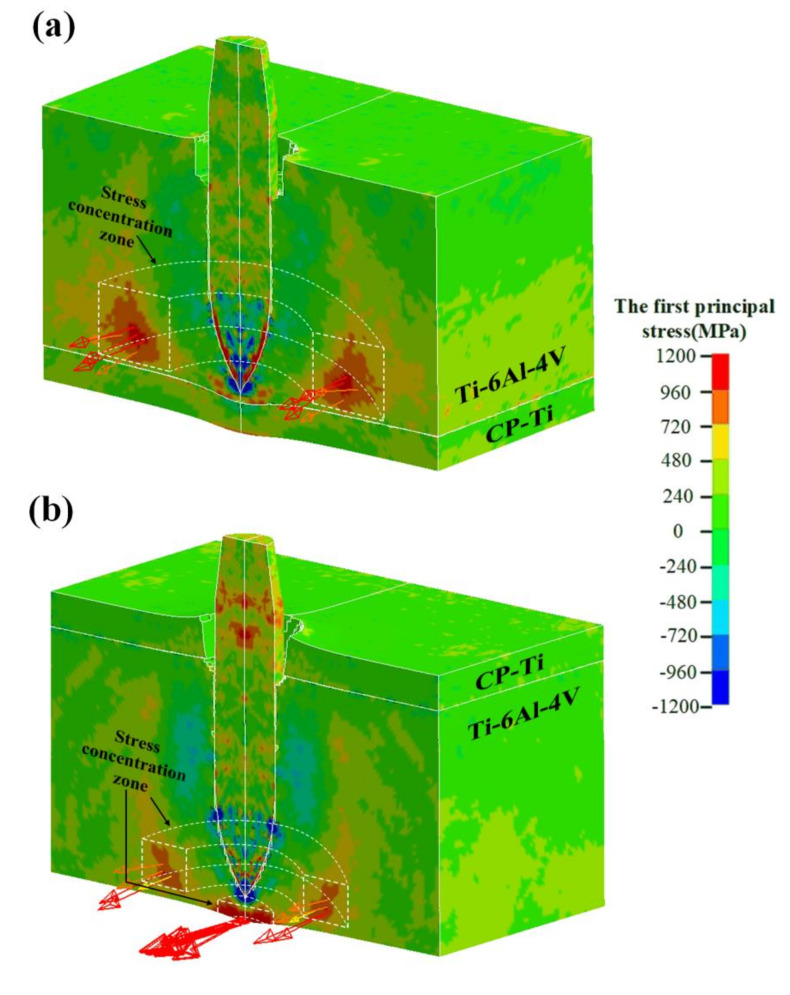
Contour of the first principal stress and the first principal stress vector of several typical elements comprising the cross-section: (**a**) Ti64/CPTi and (**b**) CPTi/Ti64.

Figure 10 shows the contour of the first principal stress associated with Ti64/CPTi and CPTi/Ti64 composite target plates at 100 µs during the penetration process. The vector of the first principal stress corresponding to several typical elements is indicated in the figure. The head of the arrow represents the direction of the stress, and the color and length represent the magnitude. A high stress concentration region, which was indirectly connected to the penetration channel and radially distributed in space, was generated in both schemes. Moreover, the stress vector showed that the first principal stress in the high stress concentration region was tensile in both schemes. The magnitude of this stress was, for some elements, >1000 MPa, i.e., higher than the tensile strength of Ti6Al4V, and therefore, cracks were preferentially initiated in these regions. In addition, the high stress concentration region of Ti64/CPTi took up more space than in the CPTi/Ti64 plate. Consequently, the lamellar tearing generated in the CPTi/Ti64 plate was less extensive and narrower than that in the Ti64/CPTi plate (as shown in Figure 6a,c). The high stress concentration region was generated on the back of the CPTi/Ti64 target plate, and the first principal stress in this region exceeded 1200 MPa. Therefore, crack formation was induced on the rear face of the target plate (as shown in Figure 6c).

## 5. Conclusions

In this work, the effect of the layer sequence on the ballistic performance of Ti6Al4V (35 mm)/CP-Ti (5 mm) laminated composite armor against a 12.7 mm API was investigated, both experimentally and computationally. Numerical simulation results of the macro morphology and penetration depth agreed well with the experimental results. Further analysis showed that the energy absorption rate of Ti64/CPTi (4.4 × 10^7^ J/s) was higher than that of CPTi/Ti64 (3.9 × 10^7^ J/s). The ballistic resistance of Ti64/CPTi was considerably higher than that of CPTi/Ti64 because more energy was absorbed in the early stage of the penetration process, thus alleviating the damage to the rear face. In addition, a highly localized principal stress (>1000 MPa) in both arrangement sequences was radially distributed in space, leading to lamellar tearing in Ti6Al4V. The stress was mainly concentrated at the rear face when the CP-Ti was placed at the front, thereby inducing cracking and failure in that region, and lowering the overall ballistic performance. This work is expected to be helpful for the further structural design of titanium laminated composite armor.

## Figures and Tables

**Table 1:** Quasi-static tensile properties of the Ti6Al4V and CP-Ti.

Materials	Yield Strength (MPa)	Ultimate Tensile Strength (MPa)	Elongation (%)
Ti6Al4V	920	974	15
CP-Ti	285	428	37

**Table 2:** Material constants of the steel core, lead jacket, and brass jacket.

Material	E (GPa)	ρ (g/cm^3^)	υ	$\sigma_{0}$ (MPa)	G (GPa)	C	P
steel	210	7.85	0.33	1200	80.0	100	10
lead	17	11.27	0.4	24	6.1	600	3
brass	115	8.52	0.31	206	44.0	-	-

**Table 3:** Johnson–Cook material constants of Ti6Al4V and CP-Ti.

Materials Parameters	Ti6Al4V	CP-Ti
A(MPa)	1098	359
B(MPa)	1092	668
n	0.93	0.49
C	0.014	0.0194
m	1.1	0.5816
D_1_	–0.09	0.5
D_2_	0.25	3.89
D_3_	–0.5	–1.74
D_4_	0.014	0.014
D_5_	3.87	0.95

**Table 4:** Experimental and simulated penetration depths.

Scheme	Experiment Penetration Depth (mm)	Simulation Penetration Depth (mm)	Relative Error
Ti64/CP-Ti	35.8	34.0	5.3%
CP-Ti/Ti64	37.1	36.8	0.8%
